# Implementation of a National Pediatric Cancer Registry in Ethiopia: A systems (“A3”) thinking approach

**DOI:** 10.1371/journal.pone.0345608

**Published:** 2026-08-19

**Authors:** James B. Collins I. V, Mandee Lines, Callee Brooks, Abdulkadir Mohammed, Daniel Hailu, Haileyesus Adam, Atalay Mulu Fentie, Mulugeta Ayalew, Diriba Fufa, Mohammed Mustefa, Tadele Hailu, Mamude Dinikiye Ali, Michael Chargualaf, Vanessa Ayer Miller, Megan Roberts, Kaitlyn Buhlinger, Benyam Muluneh

**Affiliations:** 1 UNC Eshelman School of Pharmacy, University of North Carolina, Chapel Hill, North Carolina, United States of America; 2 University of North Carolina Medical Center, Chapel Hill, North Carolina, United States of America; 3 Tikur Anbessa Specialized Hospital, Addis Ababa, Ethiopia; 4 University of Gondar Teaching and Referral Hospital, Gondar, Ethiopia; 5 Jimma University Specialized Hospital, Jimma, Ethiopia; 6 Ayder Medical Center, Mekele, Ethiopia; 7 St. Paul’s Hospital, Addis Ababa, Ethiopia; 8 Daiichi Sankyo, Chapel Hill, North Carolina, United States of America; 9 College of Pharmacy and Health Sciences, Campbell University, Buies Creek, North Carolina, United States of America; 10 UNC Lineberger Comprehensive Cancer Center, Chapel Hill, North Carolina, United States of America; PLOS: Public Library of Science, UNITED STATES OF AMERICA

## Abstract

Cancer registries allow for the collection of key data associated with patient demographics, diagnoses, and treatments, making them an essential tool for research aimed to improve the quality of care in patients with cancer. Previously, a pediatric cancer registry did not exist in Ethiopia, and prevented the collection of such data. In order to address this gap, a group of oncology clinicians and implementation scientists from the University of North Carolina worked in collaboration with all five pediatric cancer centers within Ethiopia to identify barriers and facilitators associated with a piloted REDCap registry, provide necessary training, and perform workflow analyses to create one integrated process for a pediatric cancer registry. Based on these analyses, deficiencies in the current process were identified and standard operating procedures were developed for each cancer center to guide data collection. These advancements led to the launch of Ethiopia’s first pediatric hospital based cancer registry.

## Introduction

Cancer is a leading cause of death for children and adolescents globally. It is estimated approximately 400,000 children and adolescents are diagnosed with cancer annually with the most common childhood cancers: leukemias, brain cancers, lymphomas, and solid tumors, such as neuroblastoma and Wilms tumors [1]. Cancer is now curable for more than 80% of children who have access to modern treatments and robust supportive care. However, many low- and middle-income countries (LMIC) do not have adequate access to the recent advances in treatment and thus survival rates have lagged [2,3]. In Ethiopia, the eleventh most populous country in the world, there are an estimated 150,000 new cancer cases per year, and cancer accounts for 5.8% of total national mortality [4,5]. However, it is unknown within reasonable certainty how many children in Ethiopia are diagnosed, treated, and potentially survive cancer each year, creating a major gap in care.

In 2018, the World Health Organization (WHO) developed the CureAll initiative with the goal to achieve at least 60% survival for children with cancer globally while simultaneously reducing suffering for every child with cancer. The CureAll framework consists of four pillars (centers of excellence, universal health coverage, regimens for management, and evaluation and monitoring). These pillars, in addition to three enablers (advocacy, leveraged financing, and linked governance), support countries in implementing and strengthening childhood cancer programs [6]. Cancer registries, which fall under the evaluation and monitoring pillar of the CureAll framework, are an essential tool in the collection of demographics, diagnoses, and treatment decisions for cancer patients over a large population. Data collected within registries are important for understanding improvements in treatment and assessment of the impact of quality improvement initiatives. Reliance on passive, electronic registration practices to collect these data is not currently an option in Ethiopia due to the absence of electronic health records and electronic collection systems. At this time, one population-based cancer registry exists in Ethiopia, to collect data only for patients cared for in Addis Ababa [7]. This registry is limited to patients treated within the capital city and focuses on adult patients with cancer.

There are currently five hospitals in Ethiopia that treat the vast majority of patients with pediatric cancer: Tikur Anbessa Specialized Hospital (TASH), Jimma University Specialized Hospital, University of Gondar Teaching and Referral Hospital, St. Paul’s Hospital, and Ayder Medical Center. Our collaborative team has previously documented the implementation of a pediatric oncology summary sheet (POSSh-D)—an intermediary data abstraction tool—at each of these institutions in order to facilitate the collection of demographic and diagnostic data for patients with pediatric cancer [8]. For the past several years, clinicians at each institution have utilized this form to document information from pediatric patients with cancer with variable fidelity. Data clerks have entered data from the POSSh-D into Excel spreadsheets specific to their hospital, providing institutional data [9]. Additionally, an electronic data capture application (REDCap®), which supports both online and offline data entry was utilized to develop a more secure database, and a registry was piloted at TASH. This REDCap registry pilot was adapted from the St. Jude Global Childhood Cancer Analytics Resource and Epidemiological Surveillance System (SJCARES) with some differences in collected data from the POSSh-D. However, a unified registry combining data from all 5 institutions does not currently exist. The goal of our project was to perform workflow analyses with each institution to create one integrated process for pediatric cancer data collection and establish Ethiopia’s first national pediatric cancer registry.

## Methods

### Study design

In collaboration with the pediatric hematology/oncology teams at each of the above five institutions, a team of University of North Carolina (UNC) clinicians with expertise in hematology/oncology and implementation science traveled to Addis Ababa to assist with the launch of the national pediatric cancer registry. There were three specific aims around which the methods of this implementation project were designed: 1) identify barriers and facilitators associated with the electronic REDCap survey piloted at TASH and refine the intermediate paper data collection tool (POSSh-D forms) so that these may be standardized between all institutions, 2) evaluate organizational readiness for change and provide tailored training on the registry, and 3) design site-specific standard operating procedures addressing workflow gaps (organizational and individual) in patient data collection and recording.

A modified A3 thinking strategy was used to evaluate and address the workflow gaps in patient data collection (Aim 3) [11]. A3 thinking is a structured, systems-based problem-solving methodology derived from Lean management and widely applied in healthcare quality improvement to evaluate complex workflows and identify root causes of process variation. The method emphasizes data-informed process mapping, stakeholder engagement, and iterative development of contextually appropriate countermeasures. A3 has been operationalized as a standardized, repeatable approach and its use in healthcare has been supported by studies demonstrating feasibility, consistency of application, and reliable assessment of A3 quality rather than causal effect estimation. Given the multi-site, workflow-dependent nature of registry implementation in a low-resource setting, A3 was selected as an appropriate analytic framework for Aim 3.

### Data collection

**Fig 1:**
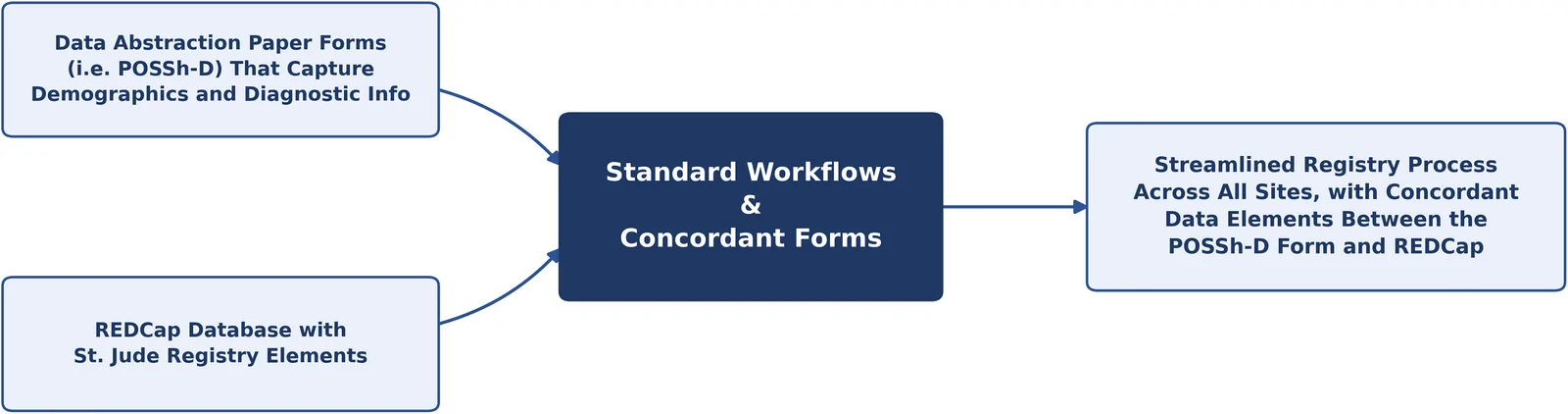
Visualization of the process used to standardize and align the data collected in the POSSh-D and REDCap Database.

The POSSh-D and SJCARES adapted REDCap registry were initially developed independently of one another. Therefore, current workflow barriers were assessed to quantify the discordance in data elements between these two tools (Aim 1) utilizing both qualitative and quantitative methods. Qualitatively, the investigators reviewed the POSSh-D and REDCap registry for consistency in the data collected, identifying any data requested in the REDCap registry that is not requested in the POSSh-D, with the goal to identify any barriers that may lead to differences in the data collected by the medical team from the data input into the registry by the data clerk. Additionally, qualitative interviews were conducted with the physicians and data entry clerks who participated in the pilot to identify barriers they faced with the current data collection system. Quantitatively, the investigators assessed the accuracy and completeness of the data entered into the REDCap pilot database from the completed POSSh-D forms. Information entered into the REDCap pilot database was assessed for completeness and accuracy when compared to the available information on the POSSh-D forms provided to the clerk by the medical team. Both the qualitative and quantitative information collected above was used to standardize workflows and make the POSSh-D and REDCap concordant to yield a streamlined registry process (Fig 1) prior to moving to Aim 2.

To assess readiness of implementing a standardized national pediatric cancer registry (Aim 2), we administered a survey including the validated Organizational Readiness for Implementing Change (ORIC) survey [10] that was amended with 3-point Likert scale response questions followed by open ended questions to better elicit perceptions of having a national pediatric cancer registry. All physicians, fellows, nurses, IT personnel, data clerks, and data analytics specialists at each of the five health systems who were involved in or responsible for pediatric cancer care or data management were invited to attend a three-day workshop focused on co-designing a sustainable workflow for the cancer registry. The ORIC survey was administered at the start of the workshop, followed by training on the importance of a national pediatric cancer registry, utilizing a POSSh-D to collect data, utilizing REDCap to upload data to the registry, and best practices for registry data quality control. A follow up ORIC survey was disseminated electronically one month after the workshop to all attendees. Likert scale responses were analyzed quantitatively. Open-ended responses were analyzed qualitatively through a consensus-based review process: two members of the study team independently reviewed all responses, identified recurring ideas, and reached agreement on key themes through discussion. Themes were not reported differentially by role given the small sample size and the primarily descriptive, consensus-building purpose of this component of the study.

### Data analysis

A3 is a structured, systems-based problem-solving methodology that emphasizes analytic process evaluation rather than inferential hypothesis testing. Analytic rigor was achieved through systematic process mapping, enumeration of workflow deviations, descriptive quantification of gaps, and consensus-based prioritization across sites.

The modified A3 process consisted of 10 steps 1) identifying/scoping the issue, 2) understanding background (i.e., context), 3) defining current condition, 4) consensus building on goals for improvement, 5) conducting root cause analyses, 6) identifying target conditions, 7) specifying countermeasures to achieve the target condition, 8) undertaking implementation and cost analysis, 9) conducting tests (i.e., measurement and evaluation), and 10) follow up/audit. The A3 analysis was conducted independently at each of the five pediatric hospitals over a two-day period.

**Fig 2:**
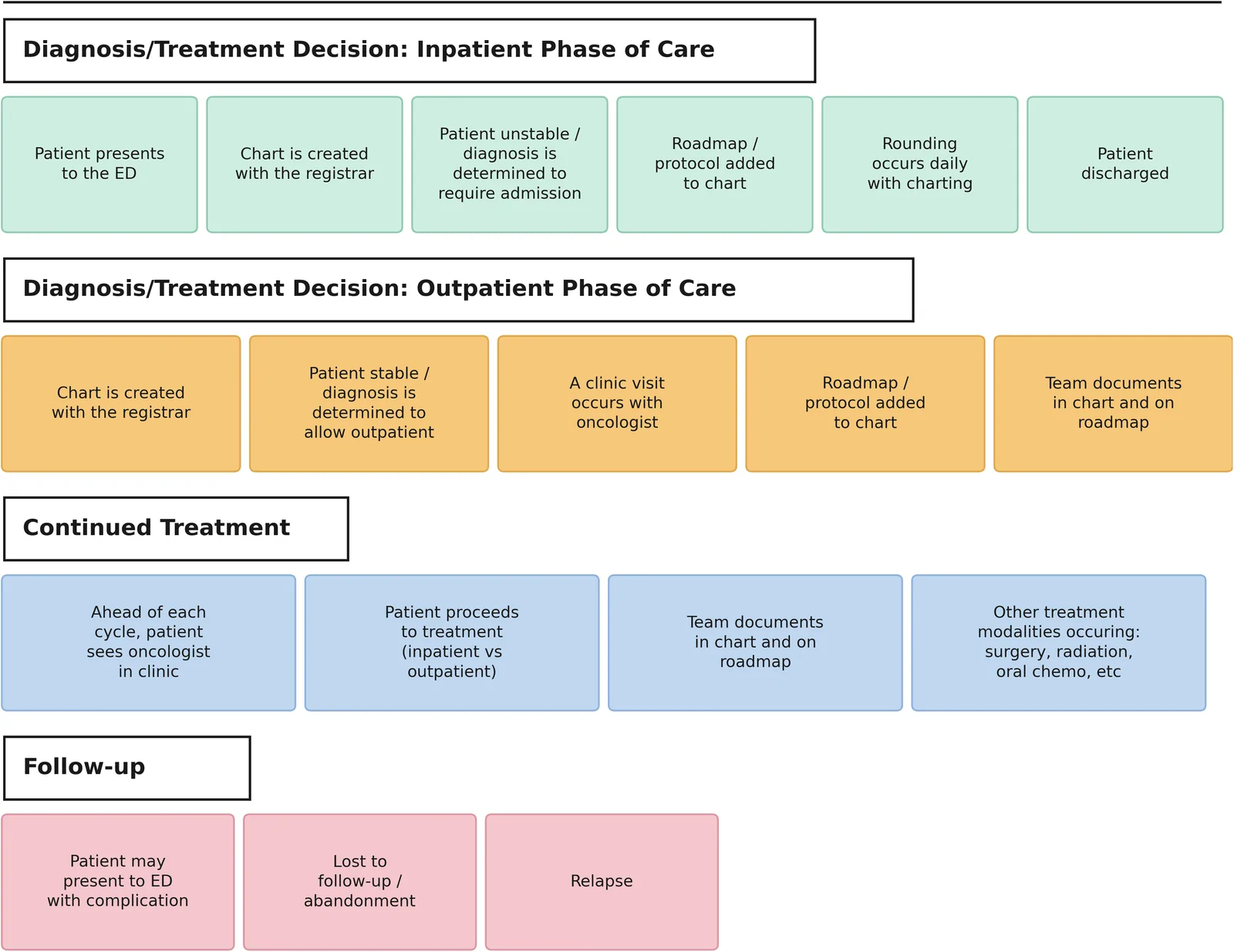
Perceived workflow used to guide discussions to identify site specific standard operating procedures for data collection for the pediatric cancer registry.

Prior to the A3 sessions, the investigators used data collected in our pre-implementation evaluation [8] to develop a conceptual model outlining the care of pediatric patients across the continuum of patient care including the diagnosis of cancer in new pediatric patients in both the inpatient and outpatient settings, throughout treatment, and long-term follow up (Fig 2). During day 1, this conceptual model was presented to representatives from each of the five hospitals for refinement and tailoring yielding site-specific workflows. Workflow analysis focused on clinical documentation processes and the physical movement of patient charts across care settings. Deviations from the refined workflows were systematically identified, enumerated, and examined to characterize gaps in routine execution of data collection processes. During day 2, discussions were centered on identifying how the POSSh-D and pediatric registry could be incorporated into each hospital’s existing site workflows. Workflow gaps were categorized, and potential countermeasures were identified through facilitated consensus. The primary analytic outputs included the number and type of workflow gaps identified, their anticipated impact on data quality, and proposed mitigation strategies with associated implementation timelines. Site-specific standard operating procedures were developed based on these analyses. Evaluation of the modified A3 focused on feasibility, internal consistency of identified gaps across sites, and generation of actionable implementation outputs, rather than causal inference. All A3 deliverables were developed collaboratively with stakeholders at each participating institution.

### Inclusivity in global research

The design and procedures for research were reviewed and approved by the investigational review board at the University of North Carolina at Chapel Hill (IRB 21–1956). This research was deemed exempt from ethical review at all institutions. Additional information regarding the ethical, cultural, and scientific considerations specific to inclusivity in global research is included in the Supporting Information (ancer registries allow for the collection of key data associated with patient demographics, diagnoses, and treatments, making them an essential tool for research aimed to improve the quality of care in patients with cancer. Previously, a pediatric cancer registry did not exist in Ethiopia, and prevented the collection of such data. In order to address this gap, a group of oncology clinicians and implementation scientists from the University of North Carolina worked in collaboration with all five pediatric cancer centers within Ethiopia to identify barriers and facilitators associated with a piloted REDCap registry, provide necessary training, and perform workflow analyses to create one integrated process for a pediatric cancer registry. Based on these analyses, deficiencies in the current process were identified and standard operating procedures were developed for each cancer center to guide data collection. These advancements led to the launch of Ethiopia’s first pediatric hospital based cancer registry.)”

## Results

### Identification of barriers during the REDCap pilot

Upon initial comparison of the POSSh-D form (data abstraction tool) and the electronic REDCap pilot registry, our team found multiple inconsistencies arising from discordant data elements. Differences between the POSSh-D and the REDCap included an undesignated calendar system on the POSSh-D (allowing some dates to be recorded using the Ethiopian calendar and others using the Gregorian calendar), open answer fields on the POSSh-D for diagnosis compared to a drop-down menus with complex lists of possible options on the REDCap pilot database, and no dedicated space for staging or treatment options on the POSSh-D despite this data being required in the REDCap. Qualitative feedback on the POSSh-D and REDCap pilot were collected from both pediatric oncology physicians and data clerks at TASH utilizing the POSSh-D and REDCap pilot (Table 1). Physicians expressed concern for the “significant number of options available [for the patient diagnosis], with many of them being adult-specific” in the REDCap. Additionally, many of the diagnoses listed required advanced diagnostic techniques that are not available to patients in Ethiopia. There were concerns from the physician group that the REDCap pilot utilized TNM staging and they “rarely use TNM staging in pediatric cancer management, unless an adult protocol was being utilized due to a lack of a pediatric protocol”. The data clerk indicated that the inconsistencies in the data collected between the POSSh-D and REDCap registry resulted in incomplete data and required them to search through the patient’s chart in order to obtain the necessary information to advance through the REDCap forms.

**Table 1:** Qualitative feedback on the POSSh-D and REDCap pilot collected from both pediatric oncology physicians and data clerks at TASH utilizing the POSSh-D and REDCap pilot.

Qualitative feedback on the POSSh-D and REDCap	
**Themes**	**Feedback received**
Reliability of REDCap	It goes down intermittently on a frequent basis. The data clerk describes one occurrence when it was down for approximately one month. Can be 1–4 days at a time.
Usability – selecting from dropdown (topography & morphology)	Fellow: it is time-consuming to select from the drop-downs, particularly while using on his phone, although it is also difficult on the desktop form. Data clerk: did not report difficulty selecting from the available drop-down options.
Usability – applicability to pediatric-specific registry	There is a significant amount of options available in drop-downs that is adult-specific. A significant amount of options do not appear to be applicable to pediatric oncology patients. Asked by providers if a significant amount of options can be removed or at least hidden to improve efficiency in entry.
Applicability	TNM staging is rarely used in pediatric cancer management (e.g., nasopharyngeal, hepatocellular). If it’s a malignancy that requires an adult protocol due to a lack of a pediatric protocol, then TNM staging may be used.
Accuracy of information entered	While reviewing each of 10 patients, it was noted that there were data points that were entered into the REDCap inaccurately (i.e., phone number placed in one of the ‘Name’ boxes, phone number that was not initially entered and was entered at time of review)
Completion	Generally a significant amount of required fields not completed in the REDCap
Standardization of workflow	There are at least 2 ongoing entries happening. The retrospective “pilot” where the providers are filling out data collection tool for the data clerk to enter into REDCap. Secondly, primarily the fellows endorse entering prospective data directly into the REDCap for patients who are presenting with new diagnoses.

Quantitatively, 10 patients out of the 343 collected in the pilot were randomly selected to evaluate for accuracy of transposed data and completeness. For these 10 patients, 118 of the 382 fields were missing (31%). The most frequently missed fields included national ID number (100% missing), address (90% missing) parent/primary caregiver email (90% missing), occupation (90% missing), phone number (90% missing), secondary caregiver email (100% missing), referral source (90% missing), date and age at diagnosis (50% missing), risk/staging information (40% missing), laterality of primary site (90% missing), start date of treatment (80% missing), and treatment protocol ID (80% missing) (Table 2).

**Table 2:** Percentage of patients where data in the POSSh-D was collected and correctly added to RedCAP registry.

POSSh-D Variable	Percent reported in RedCAP
Ethiopia Registry ID	100%
Date of Registration	100%
Hospital/Site ID	100%
Patient Name (first, middle, last)	100%
MRN	100%
Date of Birth	80%
Sex assigned at birth	100%
National ID Number	0%
Address	10%
Region	100%
Zone	90%
Country	100%
Religion	100%*
Ethnicity/Tribe	100%*
Parent/Primary Caregiver Name	100%
Phone Number	100%
Parent/Primary Caregiver Email	10%
Parent/Primary Caregiver Occupation	10%
Parent/Primary Caregiver Education	100%*
Secondary Caregiver Phone	10%
Secondary Caregiver Email	0%
HIV Status	90%*
Referral Source	10%
Does the patient have a confirmed dx?	100%
Date of diagnosis	50%
Age at diagnosis	50%
Initial diagnosis	100%
Risk/Stage	50%
Basis of diagnosis	100%
Topography (Histology)	90%
Morphology	100%
ICCC3 Code	80%
laterality of primary site	10%
Date of referral to tx institution	100%
Was tx started with curative intent	100%
Date patient started tx	10%
use of therapeutic protocol	100%
protocol ID	10%
Type of treatment started/given	60%

*Item was filled in on RedCAP but >60% of responses were marked “unknown”

### Refinement of POSSh-D form and REDCap database

Based upon the received feedback, significant refinements were made to both the POSSh-D form and the REDCap database. Changes to the POSSh-D included clarification on the use of the Ethiopian calendar for dates [12] (i.e., modified-Julian instead of Gregorian system) and updating address fields to reflect local nomenclature. Fields such as “distance from residency to treatment center” and “how long to walk to transportation” were added to help further assess a patient’s ability to obtain treatment. The diagnosis and staging sections of the POSSh-D were significantly updated to match the REDCap registry and simplify the data collection process with the POSSh-D. Open answer fields for patient diagnosis and stage were replaced with a diagnosis table to standardize the process of filling out the POSSh-D form. The diagnosis table contains the 15 most common pediatric oncologic diagnoses, as well as an option for rare diagnoses. Within each type of cancer, further options for staging, risk category, and location (for solid tumors) are provided for selection. The table allows the physician to quickly circle the disease, stage and/or risk, and location without having to write out all of the information on a single answer line. Following the diagnosis table, physicians were asked to identify the initial plans for treatment based on selection boxes. The open entry fields for chemotherapy received were replaced with questions asking for specific protocols that could easily be transcribed by the physicians. Options for off-protocol treatment and modifications to protocols were added to capture any changes to the recommended protocols.

**Fig 3:**
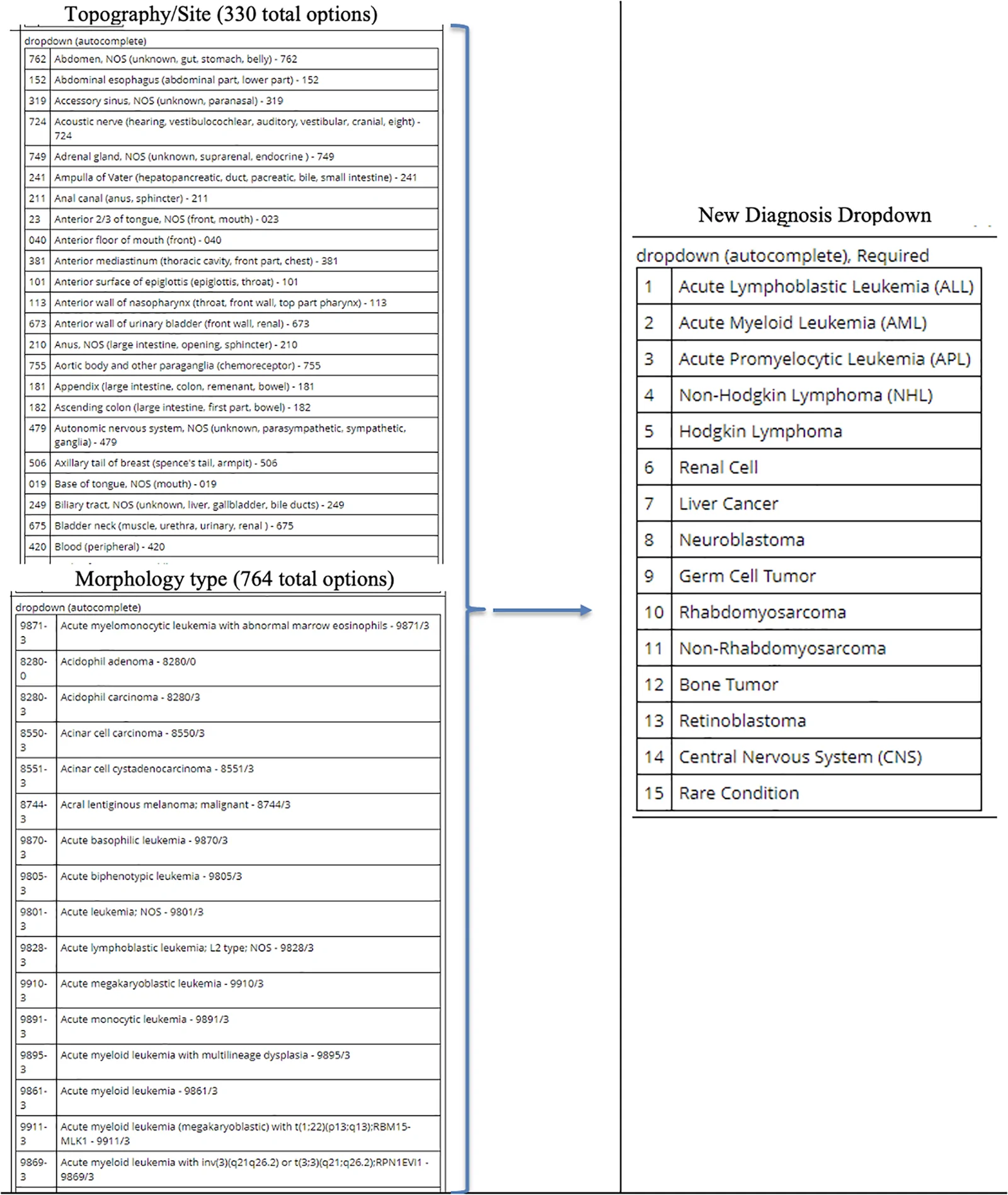
Revised registry functionality for documenting diagnoses.

Additional refinements were made to the REDCap in alignment with the changes made to the POSSh-D. Question fields in the REDCap were ordered to duplicate the exact order that the questions appeared on the POSSh-D form to help simplify the data transfer process for the data clerk (i.e., to facilitate more accurate data entry from the data abstraction form to the electronic registry). Within the REDCap, the diagnosis drop down menu was simplified from 330 topography options and 764 morphology options to the 16 options from the diagnosis table on the POSSh-D form. Once a cancer type is selected, branching logic within the REDCap database allows for collection of disease specific factors including the related risk, staging, location, and anticipated treatment regimens (Fig 3). These changes to the diagnosis section of the REDCap were adapted from the African Cancer Registry Network’s Childhood Cancer Staging Rules [13]. The updated version of the REDCap database requires all questions to have an answer choice selected in order to move onto the next questions within the database. All multiple choice or select all answer fields have an option for “none” or “missing information” so fields are not left blank.

### Readiness to implement registry

During the development and implementation of the pediatric cancer registry, representatives from each cancer center indicated that a lack of understanding of the purpose of the registry by all team members and inadequate training could impact the implementation of the registry. Therefore, a 3-day workshop and training was provided, with the ORIC survey completed to measure the effectiveness of the workshop. A total of 26 individuals participated in the ORIC survey prior to the workshop. Participants included physicians (n = 13), nurses (n = 2), pharmacists (n = 1), data entry clerks (n = 4), information and communication technicians (n = 4), health informaticists (n = 1), and community mobilization officers (n = 1). A total of 14 individuals completed the ORIC survey for a second time, one month after completion of the workshop. Participants included physicians (n = 10), information and communication technicians (n = 2), health informaticist (n = 1), and an unspecified category (n = 1). Quantitative results from the ORIC are displayed in Table 3. Overall, there was high readiness by hospital staff to adopt and launch the pediatric cancer registry, both prior to and after the workshop, with 100% of respondents indicating that they thought it is important and worthwhile to implement the cancer registry. Mean agreement with the 8 questions measuring readiness to implement change ranged from 2.48 to 2.92 prior to the workshop and 2.69–2.86 when remeasured one month after the workshop. No participants in the post-workshop survey marked a 1 (not ready to implement change) for any of the questions surveyed.

**Table 3:** Organizational Readiness for Change (ORIC) Survey Results.

Question	Pre-workshop mean (std deviation) response (n = 26)	1-month post-workshop mean response (n = 15)
**Do you think it is important/worthwhile to implement the cancer registry?**	100% yes	100% yes
**How committed is your facility to implementing this change?**	2.88 (0.32)	2.86 (0.35)
**How motivated is your facility to implementing this change?**	2.85 (0.36)	2.71 (0.45)
**How willing is your facility to work hard to implement this change?**	2.92 (0.27)	2.86 (0.35)
**How much does your facility want to implement this change?**	2.92 (0.27)	2.79 (0.41)
**How confident are you in effectively using the resources that are currently available to implement the program?**	2.48 (0.64)	2.69 (0.49)
**How confident are you in effectively supporting clinicians as they adjust their clinical practice in response to this program?**	2.76 (0.43)	2.71 (0.45)
**How confident are you in effectively solving problems that might arise in implementing this program?**	2.65 (0.55)	2.69 (0.46)
**How confident are you in effectively coordinating the efforts of those involved in implementing this program?**	2.73 (0.52)	2.71 (0.45)

Qualitative data collected through the ORIC further illustrated the desire for a pediatric registry. Participants viewed the registry as an important initiative to help advance pediatric cancer outcomes, stating, the data in the registry would allow them “to assess and improve care” as well as to identify “the patterns of cancer and lead [to]… proper planning and appropriate management of patients.” Respondents also stated both before and after the workshop that there was commitment from technical staff, health workers, and leadership. Several sites identified potential to integrate the REDCap registry with other technologies they have started to adopt, such as electronic health records, further simplifying the process. The main barrier to confidence in adoption of the registry was limited resources. Participants indicated they may be limited in adopting the registry by limited documentation, staff, and budget, leading to increased workload for those working with the registry.

### Workflow and gap analysis

Prior to the workshop, representatives from each pediatric cancer center indicated that a lack of standardized and organized process to develop the implementation blueprint, ensuring data quality, and maintaining security and storage of patient data was a barrier to implementation of the pediatric registry. Therefore, a two-day workflow analysis was completed to adapt each site’s current workflow to incorporate the POSSh-D and REDCap registry. On day 1 of the workflow analysis, there were several site-specific deviations from the assumed workflow in Fig 2. that were identified, including some sites utilizing electronic medical records, some sites admitting all patients at diagnosis while others treating stable patients in the outpatient setting, different means of transporting patients’ charts between units, and different locations for storage of the POSSh-D. Therefore, tailored standardized operating procedures were created uniquely for each participating hospital to account for differences in their current workflow. In day 2 of the workflow analysis, site specific workflows were further optimized to address responses to the following seven questions: 1) Where will blank POSSh-D forms be stored? 2) When will the POSSh-D be inserted into the chart and by whom? 3) When will information be entered into the POSSh-D and by whom? 4) How will the POSSh-D get into the hands of the data clerk when complete? 5) When will information from the POSSh-D be entered into REDCap?, 6) Will the completed POSSh-D be stored in the chart or elsewhere? and 7) How will POSSh-D be moved between phases of care (i.e., inpatient, outpatient, emergency room) and by whom?

Throughout discussions, a total of 14 gaps were identified that were not accounted for during the previous workflow. Gaps included inadequate training on data collection and input into the REDCap registry, inadequate tools to identify duplicate entries, infrastructure limitations including unreliable internet and power, and lack of guidance on maintaining the accuracy of the data in the registry long term among other things. Solutions to these gaps, as identified by the representatives from each cancer center, included development of videos for training residents and data clerks on the use of the POSSh-D and REDCap, automated warnings in REDCap and dated POSSh-Ds to prevent duplication of entry, use of the REDCap phone app to overcome internet and power outages, secure and long-term storage of completed POSSh-Ds to serve as a physical backup to the registry, and incorporation of quality control procedures to maintain accuracy of the registry. Timelines for addressing these gaps ranged from at registry launch to 6 months post launch. A comprehensive summary of these gaps and recommendations to address them are listed in Table 4. Each gap was categorized by the time frame in which it would be addressed. Verbal consensus was obtained from representatives from all five cancer centers regarding this plan to address these gaps.

**Table 4:** Identified gaps and proposed solutions prior to launch of registry.

Gaps	Recommendation/Action	Responsible Party	Timeline
Training for residents on POSSh-D	Develop video-based training for residents (will be posted on YouTube)	UNC Team	3 month
Training for data clerks on REDCap	Develop video-based training for residents (will be posted on YouTube)	UNC Team	3 months
Duplication entry	Use automatic REDCap ID number	Data Manager	At go-live
	Warning alert if first name, fathers name and date of birth match	Data Manager (IT Expert)	3 months
	(TASH) Stamp the paper chart to designate completion of POSSh-D entry into REDCap; (Gondar) change discharge note template to check box that POSSh-D done	Oncology fellow	3 months
Presumed diagnosis may change	POSSh-D can be edited once confirmed diagnosis is known. Finalized POSSh-D should have accurate information.	N/A	N/A
Getting POSSh-D to data clerk	A centralized location will be determined by each site for pediatric hem/onc fellows to drop off completed form	Peds hem/onc fellows	At go-live
Internet connection may not be reliable	Use mobile phone offline and synchronize data whenever wifi is available.	Data clerks	At go-live
Backup of REDCap	Paper version of POSSh-D should be kept securely by data clerk (and scanned into electronic record or copy into paper chart whenever possible)	Data clerks	At go-live
Gregorian vs. Julian (Ethiopian) Calendar	We will use the Ethiopian calendar on POSSh-D and data clerk will convert date into Gregorian calendar for REDCap https://ethiopian-calendar.netlify.app/	Data clerk	At go-live
Chart going home with patient (TASH)	Explore options to hire porter	Dr. Getasew/Sr. Aster	6 months
Accuracy of data collection	Quality control procedures	TBD (need to be designated)	At go-live
Lack of clarity on who should have data entry access	Consensus will be made on who should have various types of access (i.e., view, data entry, data modification, data download, REDCap form design)	Registry advisory panel	1 month
Missing data	Quality control procedures (use best judgement in completing REDCap form)	TBD (need to be designated)	At go-live
Nutritional Index Status	Add to POSSh-D and REDCap	UNC Team	Done
Development of POSSh-T and F	Co-develop POSSh-T/F with advisory panel; provide a prototype to review	Registry advisory panel	9 months

## Discussion

Our team worked closely with the five pediatric cancer centers in Ethiopia and the Ethiopian Federal Ministry of Health to successfully identify barriers to the collection of data for a national pediatric cancer registry, create standardized workflows for the registry, and launch the first national pediatric cancer registry in Ethiopia. This registry was initially piloted at a single center (TASH), and refined to improve functionality and relevance to Ethiopian patients at all five hospitals. Once refined, health care personnel underwent a 3-day workshop, where they were provided with training on how to record and upload data to the registry based on their individual roles. Lastly, through discussions with key partners, contextually-relevant workflows were designed to overcome the unique challenges present at each site. Throughout this process, individuals at all sites indicated high readiness to adopt the registry. An analysis in 2019 previously established that there was increased readiness to adopt the registry after the establishment of the POSSh-D^8^. Since this analysis, the team has been in continued virtual communication with representation from each of the pediatric cancer centers to lay the ground work for this workshop and create deliverables including a data use agreement, data governance structure, identification of key team members, and commitment from the ministry of health. Therefore, a high readiness to adopt the registry was anticipated prior to this particular workshop. This registry will provide key data surrounding the diagnosis of pediatric cancer, which may help guide the development of future health care programs in Ethiopia.

There are currently 29 countries in Africa that have population based pediatric cancer registries; 23 have registered as part of the African Cancer Registry Network (AFCRN) [14] while 6 countries have adopted the St Jude Childhood Cancer Analytics Resource and Epidemiological Surveillance System (SJCARES) [15]. Additionally, the AFCRN collects data on pediatric patients treated at TASH in Ethiopia, previously providing important information on patients treated at this one site [16]. Given the potential for multiple future registries both in Ethiopia and East Africa, Ethiopia’s National Pediatric Cancer Registry was designed to include many of the data points collected in these other registries so that data can be combined and analyzed together in future regional analyses. As these registries continue to develop, changes to the data points collected will need to be addressed thoughtfully considering site-capacity and existing workflows, tailoring the information obtained based on resources available at different LMIC sites.

In order to create a sustainable pediatric cancer registry, it was important to take a systematic approach to the adoption, implementation, and maintenance of the registry long term. During the development and implementation of the pediatric cancer registry, several barriers were foreseen that may impact the long-term viability of registry, including a lack of understanding of the purpose of the registry by all team members, inadequate training, a lack of standardized and organized process to develop the implementation blueprint, ensuring data quality, and maintaining security and storage of patient data. These barriers were addressed and mitigated through specific strategies including: a refined REDCap Pediatric Cancer Registry, a refined POSSh-D data abstraction form, training for staff and clinicians, and tailored standard operating procedures for each site. The development of each of these aspects of the registry directly addressed perceived barriers to registry success, and were achieved by directly incorporating feedback from the end users at each hospital site that eventually led to full adoption of the pediatric registry by all five hospitals.

The findings from this implementation process directly inform the next steps for sustaining and expanding the registry. The 31% overall missing data rate observed in the REDCap pilot, particularly for fields such as laterality of primary site (90% missing), treatment start date (80% missing), and protocol ID (90% missing), underscores the importance of the concordance work between the POSSh-D and REDCap undertaken in Aim 1. The redesigned diagnosis table and streamlined REDCap branching logic directly address these gaps by reducing cognitive burden at the point of data entry. Going forward, the video-based training materials planned for residents and data clerks (Table 4) will be critical to sustaining data completeness, particularly as staff turn over at participating sites. The high and sustained organizational readiness observed in ORIC scores both before and one month after the workshop—alongside qualitative expressions of commitment from technical staff, health workers, and leadership—suggests that the registry has a strong foundation for long-term adoption. However, the qualitative data also revealed that limited resources, including documentation support, staffing, and budget, remain the primary threat to sustained implementation. Future iterations of the registry should therefore prioritize low-burden quality control procedures, leverage the REDCap mobile offline application to mitigate infrastructure constraints, and explore integration with emerging electronic health record systems at sites that are beginning to adopt them. The site-specific standard operating procedures developed through the A3 analysis provide a concrete, actionable framework for each institution to move forward, and the registry advisory panel identified in Table 4 will be central to governing ongoing data quality and access decisions.

There are several limitations to this study. The registry is currently limited to the collection of diagnostic data, which prevents the incorporation of treatment outcomes and long term follow up in current analysis of the data. We anticipate the incorporation of treatment and follow-up data in future updates to the registry.

Overall, our team in collaboration with TASH, Jimma, University of Gondar, St. Paul, and Ayder has successfully launched a pediatric registry in Ethiopia. There is high readiness from all involved to adopt and implement the registry. Continued efforts will be needed to reassess the quality of the registry and continue the refinement of the registry moving forward.
